# Optimized analytical segmented method for improved intravoxel incoherent motion parameter extraction in low‐perfused tissues

**DOI:** 10.1002/mrm.30636

**Published:** 2025-07-17

**Authors:** Erick O. Buko, Suhail P. Parvaze, Casey P. Johnson

**Affiliations:** ^1^ Department of Veterinary Clinical Sciences University of Minnesota St. Paul Minnesota USA; ^2^ Center for Magnetic Resonance Research University of Minnesota Minneapolis Minnesota USA

**Keywords:** bone marrow, diffusion, intravoxel incoherent motion, magnetic resonance imaging, perfusion

## Abstract

**Purpose:**

Intravoxel incoherent motion (IVIM) diffusion‐weighted imaging of tissue perfusion and diffusion has limited signal in low‐perfused tissues, which poses a challenge in measuring IVIM parameters. This work proposes an optimized analytical segmented (opAS) method for extracting IVIM parameters and compares its performance with prior segmented (S), oversegmented (OS), and analytical segmented (AS) fitting methods.

**Methods:**

The four fitting methods were compared using numerical simulations and retrospective analysis of IVIM data of a piglet model of bone marrow ischemia. The simulations compared the accuracy and precision of the four methods for different combinations of IVIM perfusion‐related parameters (D*, pseudo‐diffusion coefficient; and *f,* pseudo‐diffusion fraction) at a fixed molecular‐diffusion coefficient (*D*). In vivo data from *n* = 11 piglets were analyzed using the four methods to compare their sensitivities in detecting lack of perfusion in ischemic versus perfused pairs of femoral heads.

**Results:**

In simulation, opAS had the lowest errors in estimating *D*, *f*, D*, and *f* × D* across all signal‐to‐noise ratio (SNR) levels, particularly in challenging parameter combinations of low *f* and D*. In the piglet model, all four IVIM fitting methods consistently found that *D* increased and *f* and *f* × D* decreased in the ischemic versus perfused femoral heads. However, D* was only consistently decreased in the ischemic versus perfused femoral heads using the AS and opAS methods.

**Conclusion:**

The opAS method more accurately estimates IVIM parameters than the S, OS, and AS approaches, particularly perfusion‐related parameters (D* and *f*) in low‐perfused tissues.

## 1 INTRODUCTION

Intravoxel incoherent motion (IVIM) is a noninvasive imaging technique that evaluates tissue microstructure and perfusion by modeling diffusion‐weighted MRI signals as a combination of diffusion and perfusion effects within each voxel.^1^ IVIM involves acquiring multiple diffusion‐weighted images at various *b*‐values (diffusion‐weighting strengths) and fitting these signals to a bi‐exponential model to extract parameters representing molecular diffusion due to the Brownian motion of water in both tissue and vascular compartments and fast pseudo‐diffusion attributed to microcirculation (blood perfusion).^1^ By quantifying the molecular‐diffusion coefficient (*D*) and the perfusion‐related pseudo‐diffusion coefficient (D*) and pseudo‐diffusion fraction (*f*), IVIM provides valuable insights into tissue structure and function.^2^, ^3^ These parameters are sometimes referred to by alternative terms, such as ADC_slow_ (or *D*_s_) for *D* and ADC_fast_ (or *D*_f_) for D*.

IVIM has demonstrated significant utility in highly perfused tissues and organs, offering improved diagnostic and prognostic capabilities across various pathological conditions.^4^, ^5^, ^6^ However, its application to low‐perfused tissues, such as bone marrow, presents challenges due to inherently weak perfusion signals.^7^ Accurate quantification of IVIM parameters in such contexts requires robust postprocessing algorithms to ensure reliable results, which are crucial for advancing characterization of tissue microstructure and perfusion.

Several methodologies have been proposed to improve estimation of IVIM parameters in low‐perfused tissues (i.e., low D* or *f*).^7^, ^8^ These methods are based on nonlinear least squares or Bayesian approaches.^8^, ^9^, ^10^, ^11^, ^12^ Although Bayesian approaches can produce visually appealing IVIM parameter maps, they may mask tissues features of interest compared to nonlinear least squares appraoches.^11^ Current nonlinear least squares approaches are based on a partial fitting techniques that assume the perfusion effect is negligible at relatively high *b*‐values, as D* is considered to be at least one order of magnitude greater than *D*.^13^ As such, beyond a given *b* threshold (*b*_t_), a mono‐exponential function can be used to extract *D*, which are then fixed while determining D* and *f*. Several partial fitting methods have been developed, including the segmented (S), oversegmented (OS), and analytical segmented (AS) fitting approaches.^8^, ^14^, ^15^ These methods have demonstrated their ability to detect physiological differences in various tissues, underscoring their potential clinical relevance.^15^, ^16^ However, their performances hinge on critical factors, notably the selection of *b*_t_ used in calculating *D*.^17^, ^18^ Any errors in the measurement of *D* propagate into the estimation of D* and *f*, which can compromise the accuracy of IVIM and limit its utility.

This study aimed to address these challenges by proposing an optimized analytical segmented (opAS) method for extracting IVIM parameters in low‐perfused tissues. We developed and validated the opAS method through numerical simulations and in vivo imaging of perfused versus non‐perfused femoral heads in a piglet model of bone marrow ischemia. The accuracy and precision of the opAS method were compared with other partial fitting approaches to validate opAS as a robust and optimized IVIM postprocessing technique to advance research and clinical application of IVIM imaging, particularly in challenging low‐perfusion tissue environments.

## 2 METHODS

### 2.1 Theory

IVIM models diffusion signal attenuation due to the application of diffusion weightings (*b*‐values) as a two‐compartment model representing tissue and vascular compartments as follows^16^, ^19^:

(1) $$S\left(b\right)=S\left(0\right)\left[fe^{-b\left(D+D^{*}\right)}+\left(1-f\right)e^{-\mathrm{bD}}\right]$$

where *b* is the diffusion weighting; *D* is the molecular‐diffusion coefficient associated with Brownian motion in both tissue and vascular compartments; D* is the pseudo‐diffusion coefficient attributed to microcirculation in vascular compartment; and *f* is the pseudo‐diffusion fraction. The segmented (S), oversegmented (OS), analytical segmented (AS), and proposed optimized analytical segmented (opAS) approaches to extract IVIM parameters (*D*, D*, and *f*) are described subsequently and summarized in Figure S1.

### 2.2 Segmented (S) approach

The S method is a partial fitting approach that relies on the assumption that signal contributions due to pseudo‐diffusion are negligible at b‐values higher than a given threshold (*b*_t_).^11^, ^13^ Thus, *D* can be extracted by fitting diffusion‐weighted imaging signals using a mono‐exponential function for *b*‐values greater than *b*_t_ (typically ranging from 20 to 200 s/mm^2^), as follows^15^, ^17^, ^18^, ^20^:

(2) $$S\left(b\right)=S_{\mathrm{int}}.e^{-\mathrm{bD}};b\geq b_{t}$$

where *S*_int_ is the fitted y‐intercept assuming no pseudo‐diffusion component. To obtain D* and *f*, Eq. (1) is fitted using all b values with the *D* obtained from Eq. (2).

### 2.3 Oversegmented (OS) approach

The first step of the OS method^13^ is same as the S method to obtain *D* and *S*_int_ (Eq. [2]). The *f* value then is calculated as follows:

(3) $$f=1-S_{\mathrm{int}}/S\left(0\right)$$

D* is calculated by fitting Eq. (1) using all *b* values with *D* and *f* obtained from Eqs. (2) and (3), respectively.

### 2.4 Analytical segmented (AS) approach

As with the S and OS methods, the first step of the AS method^8^, ^15^ is to calculate *D* using Eq. (2). To obtain the pseudo‐diffusion parameters, Eq. (1) is first rearranged, as shown in Eq. (4), which can then be rewritten as Eq. (5):

(4) $$1-\frac{S\left(b\right)}{S\left(0\right)}e^{\mathrm{bD}}=f\left(1-e^{-bD^{*}}\right)$$

(5) $$\tilde{f}\left(b\right)=f_{0}\left(1-e^{-bD^{*}}\right)+\varepsilon$$

where *f*_0_ is the asymptotic value of the pseudo‐diffusion fraction independent of *b*‐value, and *ε* accounts for error in the measurement of *S*(0). Note that the inclusion of ε is a modification to the original description of the AS method,^8^, ^15^ which we introduced here to improve the robustness in estimating pseudo‐diffusion parameters (*f* and D*). Using *D* from Eq. (2), the left‐hand side of Eq. 4 is calculated for every *b*‐value to obtain $\tilde{f}\left(b\right)$. With these $\tilde{f}\left(b\right)$ values, Eq. (5) is fitted to extract *f*_0_ and D* using all acquired *b*‐values. Note that *f*_0_ is the measured pseudo‐diffusion fraction using the AS approach.

### 2.5 Optimized analytical segmented (opAS) approach

The opAS method aims to optimize the estimation of *D* independent of *b*_t_ before calculating the left‐hand side of Eq. (4) to get $\tilde{f}\left(b\right)$. The method involves three steps. First, if *b*_t_ is taken to be 0 (i.e., assuming no detectable pseudo‐diffusion), then IVIM can be assumed to model a mono‐exponential decay curve with an apparent diffusion coefficient (ADC) as a decay constant. Equating IVIM (Eq. [1]) to this mono‐exponential decay curve, we obtain:

(6) $$S\left(0\right)\left[fe^{-b\left(D+D^{*}\right)}+\left(1-f\right)e^{-\mathrm{bD}}\right]=S\left(0\right)e^{-b\cdot \mathrm{ADC}}$$

Second, taking the derivative of both sides of Eq. (6) with respect to *b* at *b* = 0 (i.e., without the influence of the applied *b*‐value) yields:

(7) $$D=\mathrm{ADC}-f\cdot D^{*}$$

Assuming ADC and D* are constant, and substituting Eq. (5) into Eq. (7) with *b* replaced with b_t_ and ignoring the error term (*ε*), we obtain:

(8) $$\tilde{D}\left(b_{t}\right)=\mathrm{ADC}-f_{0}D^{*}\left(1-e^{-b_{t}\cdot D^{*}}\right)$$

A series of $\tilde{D}\left(b_{t}\right)$ is obtained using Eq. (2) by varying *b*_t_ and then fitting the right‐hand side of Eq. (8) to obtain ADC and *f*_0_ D*. Note that, here, ADC is estimated from a series of $\tilde{D}\left(b_{t}\right)$ as opposed to conventional ADC, which is extracted from a series of *S*(b). The value of *D* is then determined using Eq. (9) as *b*_t_ approaches infinity:

(9) $$D=\mathrm{ADC}-f_{0}D^{*}$$

Third, *f*_0_ and D* are estimated as in the AS approach using Eqs. (4) and (5) with the *D* value obtained from Eq. (9).

### 2.6 Numerical simulations

Guided by previous simulation and in vivo studies,^8^, ^10^, ^11^, ^15^ MRI signals were generated using Eq. (1) for tissues with a fixed molecular‐diffusion coefficient (*D* = 1.0 × 10^−3^ mm^2^/s) and different IVIM perfusion‐related pseudo‐diffusion parameter combinations (*f* ranging from 0.02 to 0.50 in steps of 0.02 and D* ranging from 2.0 × 10^−3^ to 60 × 10^−3^ in steps of 2.0 × 10^−3^ mm^2^/s). Eighteen *b*‐values (0, 20, 30, 40, …, 100, 150, 200, …, 500 s/mm^2^) were used to generate IVIM signals using Eq. (1). Rician noise was added to generate SNR levels of 10, 20, and 40 at each sample point. SNR was defined as the ratio of S(0) to the standard deviation of the noise. Each sample was simulated 1000 times. IVIM parameters were estimated using the S, OS, AS, and opAS approaches. For S, OS, and AS, two sets of IVIM parameters were estimated by setting *b*_t_ = 100 and 200 s/mm^2^.

### 2.7 In vivo 3T MRI of piglet model

We retrospectively analyzed IVIM data from an Institutional Animal Care and Use–approved piglet model study of bone marrow ischemia^15^ using the S, OS, AS, and opAS approaches. Data were included from 11 piglets that underwent surgery at 6 weeks of age to induce complete ischemia in one of their femoral heads, with the unoperated, contralateral femoral head serving as a perfused control.^15^ One week after surgery, bilateral hips of each piglet were imaged in vivo at 3T MRI using RESOLVE diffusion‐weighted imaging with a subset of 13 *b*‐values used in the simulations (*b* = 0, 20, 30, 40, …, 100, 200, 300, 500 s/mm^2^) and the following parameters: field of view = 208 × 208 mm^2^, sampling matrix = 188 × 188, spatial resolution = 1.1 × 1.1 mm^2^, slices = 15, slice thickness/gap = 2.0/0.5 mm, repetition time/echo time (TE)/TE1/TE2 = 2500/68/122 ms, echo spacing = 0.5 ms; echo‐planar imaging factor = 94, bandwidth = 578 Hz/px, readout segments = 5, and GRAPPA R = 2. Lack of perfusion (i.e., ischemia) in the operated femoral heads was confirmed with gadolinium‐based contrast‐enhanced MRI for all 11 piglets.^15^

### 2.8 Data analysis

For the simulations, estimated IVIM parameters D*, *f*, *f* × D*, and *D* were compared with true values. Normalized mean error (NME), coefficient of variation (CoV), and normalized root mean square error were calculated and compared using Eqs. (10), (11), and 12, respectively, for each parameter, as follows:

(10) $$\mathrm{NME}=\frac{\frac{1}{N}\sum_{i}^{N}\left(\theta_{i}-\theta_{T}\right)}{\theta_{T}}$$

(11) $$\mathrm{CoV}=\frac{\sqrt{\frac{1}{N}\sum_{i}^{N}{\left(\theta_{i}-<\theta >\right)}^{2}}}{<\theta >};<\theta >=\frac{1}{N}\sum_{i}^{N}\theta_{i}$$

(12) $$\text{NRMSE}=\frac{\sqrt{\frac{1}{N}\sum_{i}^{N}{\left(\theta_{i}-\theta_{T}\right)}^{2}}}{\theta_{T}}$$

where $\theta_{i}$ and $\theta_{T}$ are the estimated *i*th parameters and their true values, respectively, and *N* is the sample size.

For the in vivo data, *D*, D*, and *f* quantitative maps were generated using the four IVIM fitting approaches, with *b*_t_ = 100 s/mm^2^ for S, OS, and AS. Regions of interest constituting the bone marrow of ischemic and control femoral heads were manually drawn using a central slice of the *b* = 0 image. Median values of the IVIM parameters in the ischemic and control femoral heads were measured and compared among the S, OS, AS, and opAS approaches. We also assessed paired differences between the ischemic versus control femoral heads using paired t‐tests (*p* < 0.05).

## 3 RESULTS

### 3.1 Numerical simulations

**FIGURE 1:**
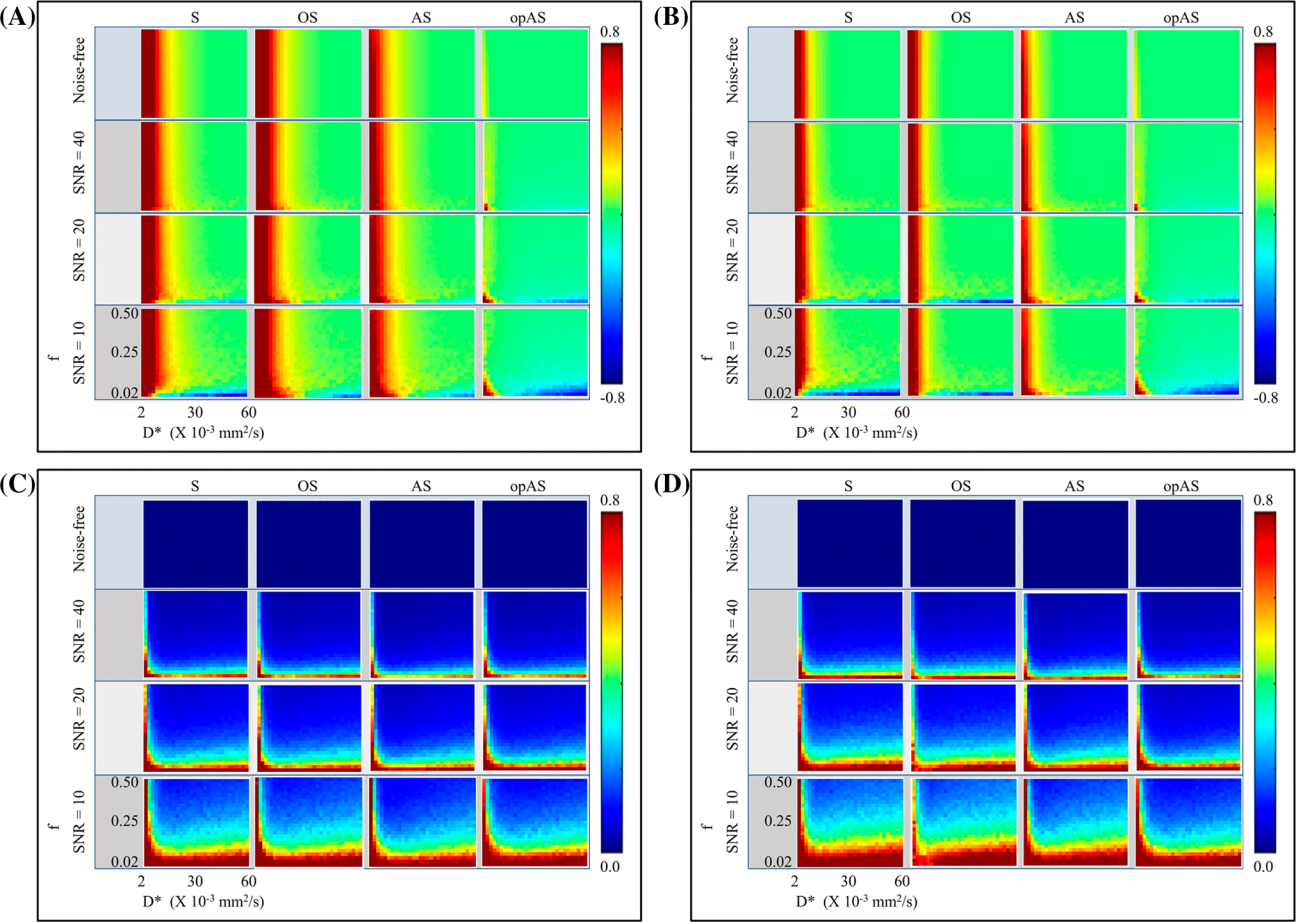
Simulated normalized mean errors (NMEs) and coefficients of variation (CoVs) of the estimated intravoxel incoherent motion (IVIM) pseudo‐diffusion coefficient (D*) using the segmented (S), oversegmented (OS), analytical segmented (AS), and optimized analytical segmented (opAS) fitting methods. The methods estimated D* at different combinations of *f* and D* at a fixed *D* value of 1.0 × 10^−3^ mm^2^/s. NMEs were measured with *b*‐threshold (*b*_t_) set to 100 s/mm^2^ (A) and 200 s/mm^2^ (B), and the corresponding CoVs are shown in (C) and (D), respectively. In the plots, *f* increases vertically, and D* increases horizontally. Of the four methods, opAS had the lowest NMEs in the estimate of D* at all regions and signal‐to‐noise ratio (SNR) levels, particularly at low D* values. Additionally, AS outperformed S and OS. For S, OS, and AS approaches, D* was highly overestimated at lower *b*_t_ (particularly at low D*). CoVs for all methods were greater at lower SNR levels (particularly at low *f*), with no differences across the methods.

**FIGURE 2:**
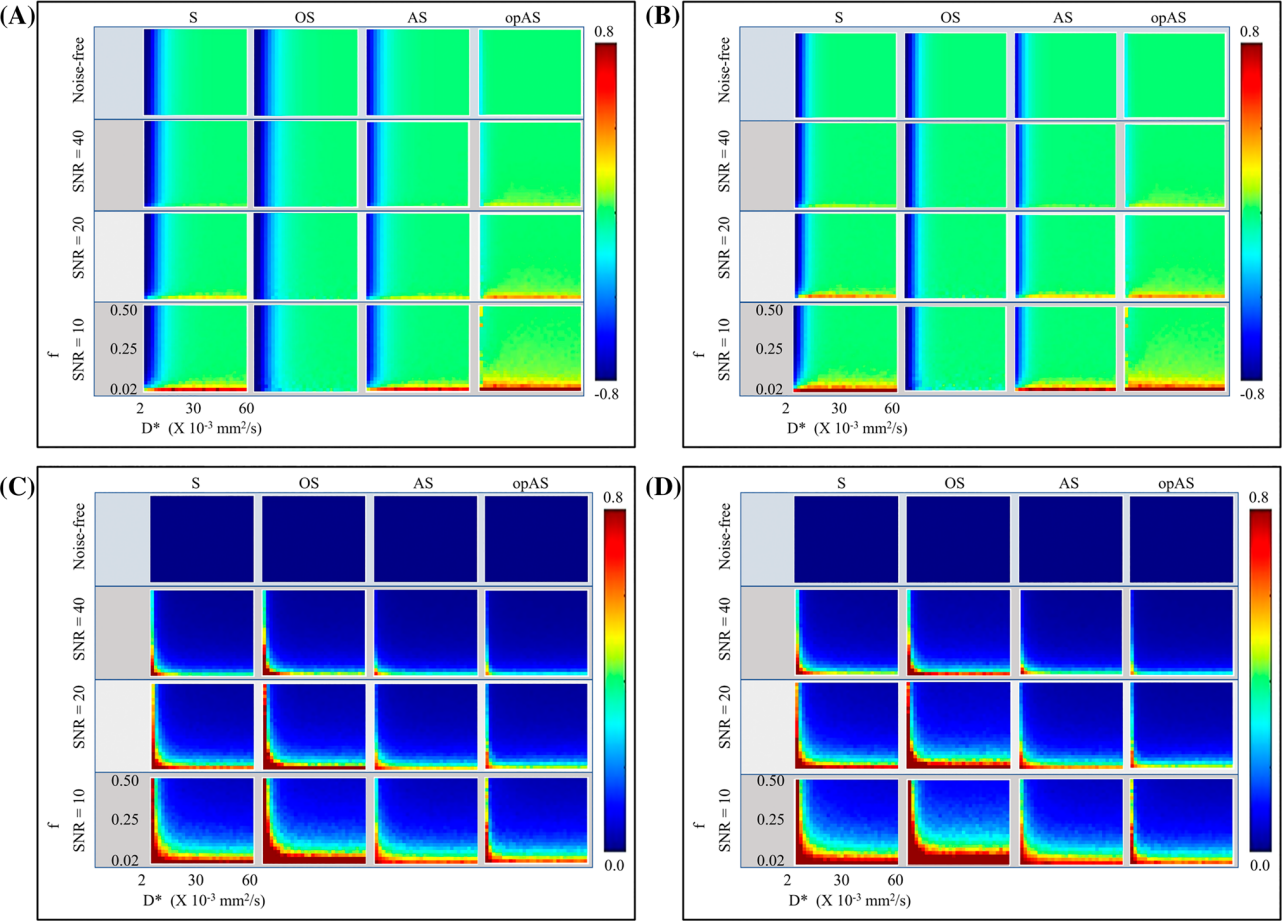
Simulated normalized mean errors (NMEs) and coefficients of variation (CoVs) of the estimated intravoxel incoherent motion (IVIM) pseudo‐diffusion fraction (*f*) using the segmented (S), oversegmented (OS), analytical segmented (AS), and optimized analytical segmented (opAS) fitting methods. The methods estimated *f* at different combinations of *f* and D* at a fixed *D* value of 1.0 × 10^−3^ mm^2^/s. NMEs were measured with *b*‐threshold (*b*_t_) set to 100 s/mm^2^ (A) and 200 s/mm^2^ (B), and their corresponding CoVs are shown in (C) and (D), respectively. In the plots, *f* increases vertically, and D* increases horizontally. Of the four methods, opAS had the lowest error in the estimate of *f* at all regions and signal‐to‐noise ratio (SNR) levels, particularly at low D*. AS outperformed S and OS. For S, OS, and AS approaches, *f* was highly underestimated at lower *b*_t_ (particularly at low D*). CoVs for all methods were greater at lower SNR levels (particularly at low *f*), with slightly lower values in AS and opAS than S and OS.

**FIGURE 3:**
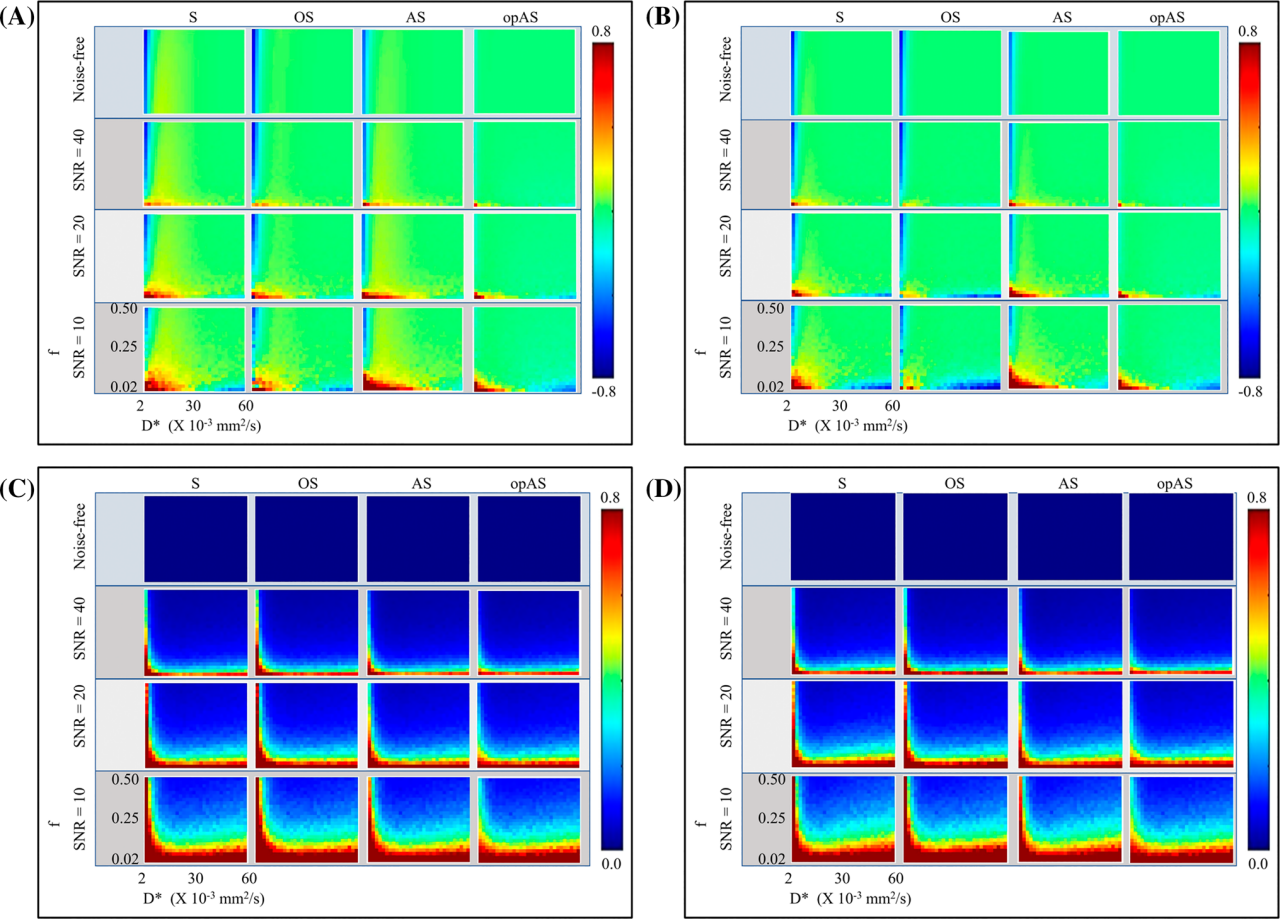
Simulated normalized mean errors (NMEs) and coefficients of variation (CoVs) of the estimated intravoxel incoherent motion (IVIM) flux (*f* × D*) using the segmented (S), oversegmented (OS), analytical segmented (AS), and optimized analytical segmented (opAS) fitting methods. The methods estimated *f* × D* at different combinations of *f* and D* at a fixed *D* value of 1.0 × 10^−3^ mm^2^/s. NMEs were measured with *b*‐threshold (*b*_t_) set to 100 s/mm^2^ (A) and 200 s/mm^2^ (B), and their corresponding CoVs are shown in (C) and (D), respectively. In the plots, *f* increases vertically, and D* increases horizontally. NMEs were similar for all methods, with a slight improvement using opAS. CoVs for all methods were greater at lower signal‐to‐noise ratio (SNR) levels (particularly at low *f*), with no differences across the methods.

NMEs and CoVs of the estimated IVIM pseudo‐diffusion coefficients (D*) are shown in Figure 1. The value of D* measured using S, OS, and AS improved in accuracy with increasing *b*_t_, particularly at low D* values. opAS outperformed the other approaches in estimating D* across the SNR range, with the most significant accuracy improvement at low D* values. All methods exhibited similar CoV across the regions and SNR levels. AS outperformed S and OS in estimating D* across the SNR range. Figure 2 shows NMEs and CoVs of the estimated IVIM pseudo‐diffusion fractions (*f*). opAS outperformed all other approaches in estimating *f*, most significantly at low D* values. The estimation of f using S, OS, and AS at low D* improved with greater *b*_t_. CoVs were lower for AS and opAS than for S and OS. Errors in the estimated pseudo‐diffusion flux (*f* × D*) (Figures 3) and molecular‐diffusion coefficient (*D*) (Figure S2) were slightly improved with opAS compared with the other approaches. Normalized root mean square errors for all four fitting methods are shown in Figure S3. The robustness of measuring the IVIM parameters decreased for all methods at lower D*, *f*, and SNR values. Overall, opAS outperformed the other approaches in estimating IVIM parameters across the SNR range, with the most significant improvement at low D*.

### 3.2 In vivo 3T MRI of piglet model

**FIGURE 4:**
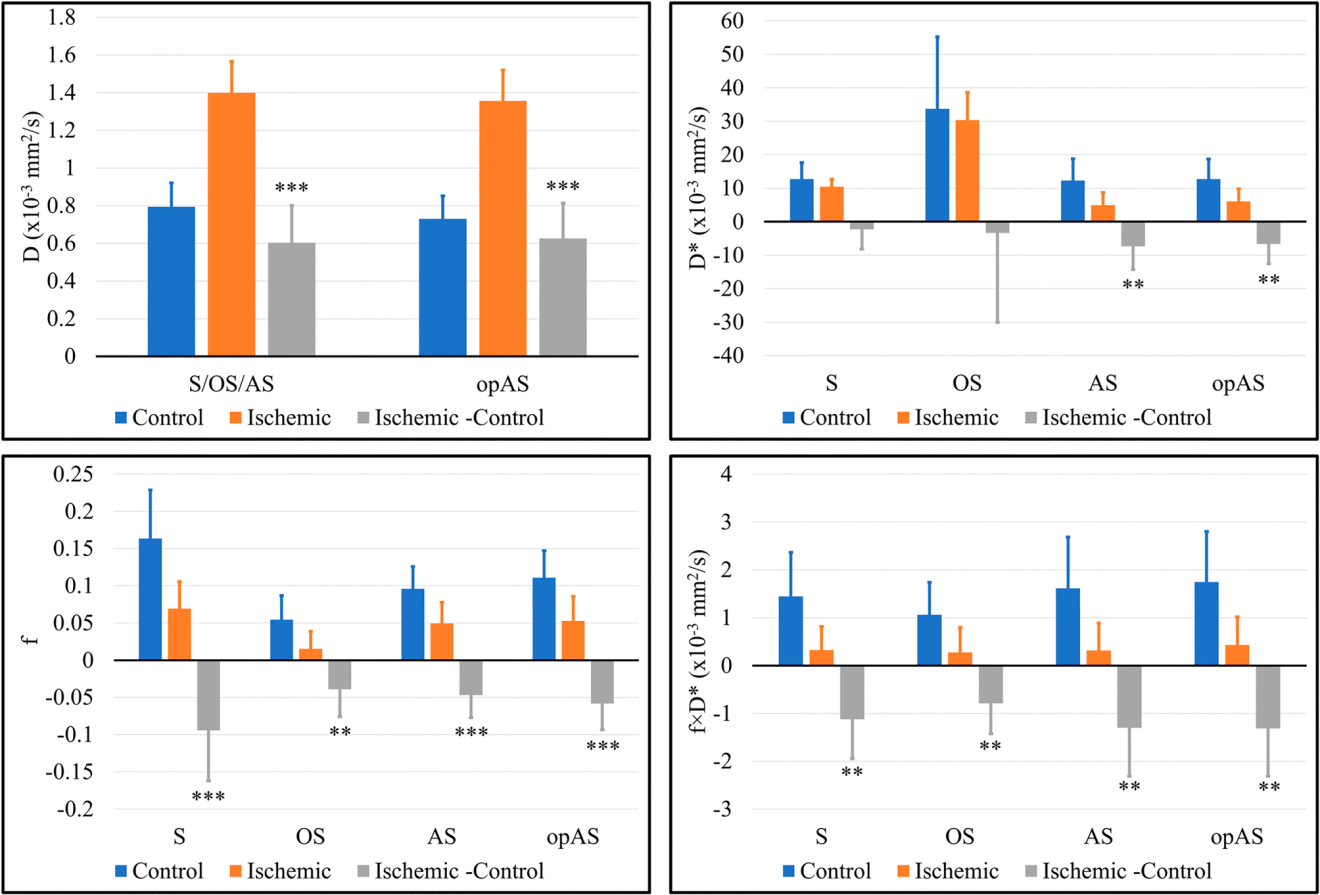
Mean and standard deviation of the estimated intravoxel incoherent motion (IVIM) parameters in *n* = 11 piglets using the segmented (S), oversegmented (OS), analytical segmented (AS), and optimized analytical segmented (opAS) fitting approaches. All four methods showed a significant increase in *D* and a significant decrease in *f* and *f* × D* in the ischemic versus control femoral heads. However, while there was no significant difference in the measured D* in the ischemic versus control femoral heads using the S and OS approaches, there was a significant decrease in D* values measured using AS and opAS. Error bars = standard deviation. *p < 0.05, **p < 0.01, ***p < 0.001.

Across all *n* = 11 piglets, all four IVIM fitting approaches showed a significant increase in *D* and a decrease in *f* and *f* × D* in ischemic versus control femoral head pairs (Figure 4). On average, *D* increased by 0.60 ± 0.20 × 10^−3^ mm^2^/s (*p* < 0.0001) for S, OS, and AS and 0.63 ± 0.19 × 10^−3^ mm^2^/s (*p* < 0.0001) for opAS. For S, OS, AS, and opAS, respectively, *f* decreased by 0.09 ± 0.07 (*p* = 0.0010), 0.04 ± 0.04 (*p* = 0.0055), 0.05 ± 0.03 (*p* = 0.0005), and 0.06 ± 0.04 (*p* = 0.009), and *f* × D* decreased by 1.12 ± 0.83 × 10^−3^ (*p* = 0.0011), 0.79 ± 0.64 × 10^−3^ (*p* = 0.0021), 1.30 ± 1.01 × 10^−3^ (*p* = 0.0016), and 1.31 ± 1.00 × 10^−3^ (*p* = 0.0014) mm^2^/s. On the other hand, while there was no significant difference in the measured D* in ischemic versus control femoral heads using S and OS (−2.27 ± 5.90 × 10^−3^ mm^2^/s, *p* = 0.23; and −3.36 ± 26.67 × 10^−3^ mm^2^/s, *p* = 0.68, respectively), there was a significant decrease using AS and opAS (7.36 ± 6.93 × 10^−3^ mm^2^/s, *p* = 0.0055; and 6.64 ± 5.92 × 10^−3^ mm^2^/s, *p* = 0.0040, respectively).

**FIGURE 5:**
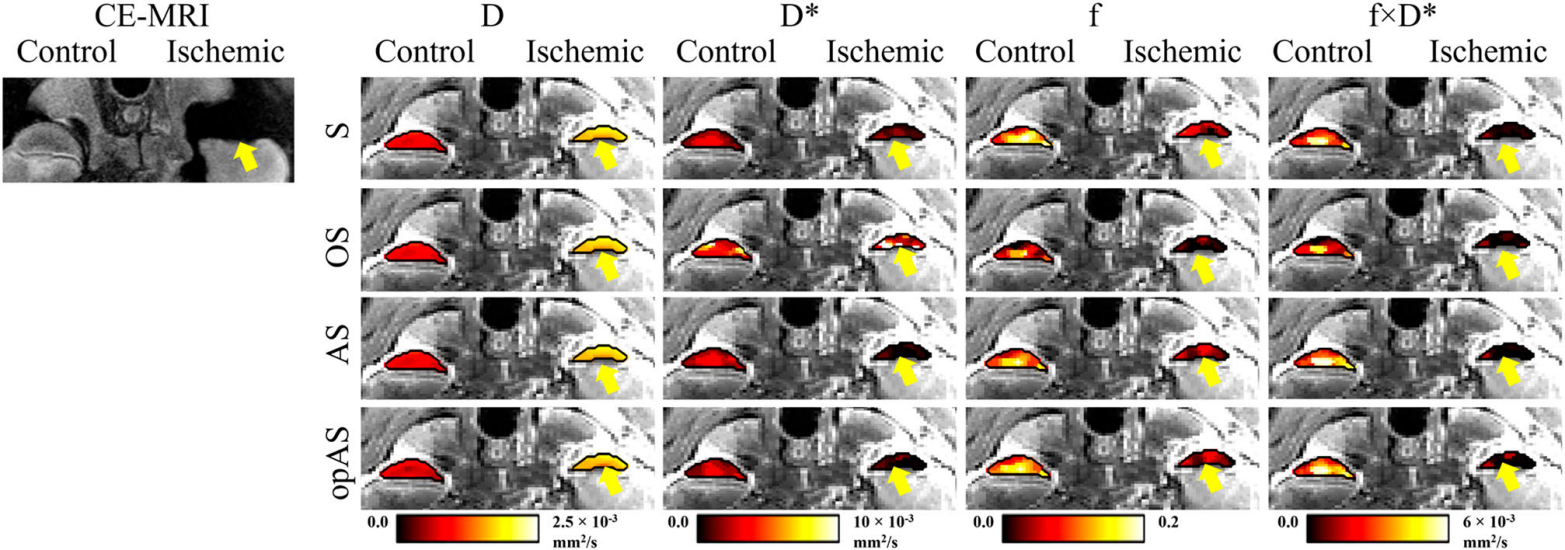
Subtracted contrast‐enhanced MRI (CE‐MRI) images and intravoxel incoherent motion (IVIM) parameter maps (*D*, molecular‐diffusion coefficient; D*, pseudo‐diffusion coefficient; *f*, pseudo‐diffusion fraction;  *f* × *D*_f_, pseudo‐diffusion flux) of the femoral heads of a representative piglet fit using the four methods: segmented (S), oversegmented (OS), analytical segmented (AS), and optimized analytical segmented (opAS). CE‐MRI confirmed complete ischemia in the operated femoral heads (*yellow arrows*). For all the fitting methods, *D* increased and *f* and *f* × *D*_f_ decreased in the ischemic versus control femoral heads. However, there was a more pronounced decrease in D* in the ischemic femoral heads using the AS and opAS methods than using the S and OS approaches.

Figure 5 shows IVIM parameter maps measured using S, OS, AS, and opAS for a representative piglet. All four fitting methods consistently showed increased *D* and decreased *f* in the ischemic versus control femoral heads. However, D* was only markedly decreased using AS and opAS. IVIM maps for a second representative piglet are shown in Figure S4.

## 4 DISCUSSION

Our simulation findings demonstrate that the proposed opAS method more accurately estimates the IVIM parameters *D*, D*, and *f* compared with S, OS, and AS approaches, particularly at low D* and *f* values. In vivo IVIM data analysis demonstrated that AS and opAS improved the distinction in D* between ischemic and control‐perfused femoral heads compared with S and OS approaches. This improvement in measurement of D* is significant and may be useful for a number of tissues for which pathological changes in D* have been challenging to detect.^15^, ^21^, ^22^ Collectively, our findings support that the opAS method provides more accurate estimates of IVIM parameters in low‐perfused tissues than prior methods.

The simulation results reveal critical limitations of the conventional S and OS methods. These methods exhibited significant inaccuracies at low *f* and D*, particularly under low SNR conditions. Such parameter combinations are frequently encountered in biological tissues, where IVIM perfusion imaging is applied to assess microcirculation. The AS and opAS methods, however, maintained relatively low errors across these regions. The opAS fitting approach provides a more accurate measurement of D* and *f* than the other methods on account of its independence of *b*_t_. opAS measures *D* as *b*_t_ approaches infinity, and D* and *f* as *b* approaches infinity, thus reducing dependency on both the chosen *b*_t_ and *b*‐values. Although AS estimates D* and *f* as *b* approaches infinity, it still depends on *D* estimated at a finite *b*_t_ (like the S and OS methods); hence, any error in the measurement of *D* using the S, OS, or AS methods propagates into the estimated pseudo‐diffusion parameters, leading to their dependency on *b*_t_. Although there is only a slight improvement in the measurement of *D* using opAS versus other the methods, opAS significantly improves estimation of D* and *f*, particularly at low *f* and D* values. Thus, inaccurate estimates of *D* could be a cause of poor reproducibility of D* with prior approaches.^14^, ^23^, ^24^, ^25^ Furthermore, our simulations showed little dependency of *f* × D* on *b*_t_, which explains its potential utility even when estimates of D* and *f* are poor.^15^ Independence of *f* × D* on *b*_t_ suggests that an underestimation of *f* is compensated by an overestimation of *D*_f_ (and vice versa). However, the improved accuracy and precision of the opAS method provide a more robust approach to estimating all IVIM parameters and avoiding potential artifactual correlations between them.

Our observed dependencies of the measured IVIM parameters on *b*_t_ using the S, OS, and AS approaches are consistent with previous studies.^17^, ^18^ For instance, a study on the upper abdominal organs found a decrease in *D* and D* and an increase in *f* with increasing *b*_t_, with greatest variation observed in D*.^17^ A similar observation was also shown in another study focused on the liver.^18^ Previous studies using the S and OS approaches have found that measurement of D* is imprecise, even in highly perfused tissues like the pancreas and liver,^26^, ^27^, ^28^ and has limited ability to distinguish physiological conditions in a number of organs.^26^, ^29^, ^30^, ^31^, ^32^ Our study indicates that the greatest *b*_t_ dependency of the estimated IVIM parameters using S, OS, and AS approaches occurs at low D* values, which is the case in low‐perfused tissues such as bone marrow. In our in vivo data analysis, D* measured using S and OS could not reliably distinguish ischemic and perfused‐control femoral heads due to overestimation of D* in ischemic (low D*) versus perfused (higher D*) femoral heads. As D* becomes low (close to *D*), its influence extends to longer *b*‐values, thus requiring a higher *b*_t_ to separate pseudo‐diffusion and molecular‐diffusion components. The use of high *b*‐value images for IVIM analysis can be challenging, as they are associated with low SNR and kurtosis effects. The opAS method mitigates these challenges by estimating *D* (and subsequently D* and *f*) without the need for very high *b*‐values. Note that the modification of the AS approach introduced herein (i.e., the error term *ε*) significantly improved the estimation of D* in the in vivo data analysis compared with what was previously reported.^15^ However, our simulation findings support that opAS is the superior fitting approach, as its independence of *b*_t_ provides more accurate quantification of IVIM parameters.

A limitation of our analysis was that too few high *b*‐value images were available in the in vivo study data to test fitting of the S, OS, and AS methods with *b*_t_ > 100 s/mm^2^. Only three *b*‐value images > 100 s/mm^2^ were acquired, whereas the simulations demonstrated that additional high *b*‐values may improve the estimation of the IVIM parameters with *b*_t_ = 200 s/mm^2^. Nonetheless, our results demonstrate that the opAS method can more accurately estimate IVIM parameters, particularly when there are practical limitations on the number of *b*‐values images that can be acquired. Further advancement of the opAS method may be possible in future investigations of the optimal *b*‐values needed for robust fitting, effects from non‐Gaussian diffusion and slow‐diffusing protons, and other imaging parameters that may affect IVIM parameter estimations, such as echo time and field strength.^33^, ^34^, ^35^, ^36^, ^37^

In conclusion, the opAS fitting approach more accurately estimates IVIM parameters than the S, OS, and AS fitting approaches, particularly the perfusion‐related parameters D* and *f* in low‐perfused tissues. Because the opAS method does not rely on defining a b‐threshold, it can potentially improve the reliability and clinical applicability of IVIM imaging across a spectrum of diseases and anatomical regions.

## Supporting information


**Figure S1.** Algorithmic steps used to extract intravoxel incoherent motion (IVIM) parameters from diffusion‐weighted MRI using the segmented (S), oversegmented (OS), analytical segmented (AS), and optimized analytical segmented (opAS) methods.
**Figure S2.** Simulated normalized mean errors (NMEs) and coefficients of variation (CoVs) of the estimated intravoxel incoherent motion (IVIM) molecular diffusion coefficient (*D*) using the segmented (S) (and equivalently oversegmented [OS] and analytical segmented [AS]) and optimized analytical segmented (opAS) fitting methods. The methods estimated *D* at different combinations of *f* and D* at a fixed *D* value of 1.0 × 10^−3^ mm^2^/s. In the plots, *f* increases vertically, and D* increases horizontally with b‐threshold (*b*_t_) set to 100 s/mm^2^ and 200 s/mm^2^. The value of opAS had lower NMEs and CoVs in the estimate of *D* at all regions and signal‐to‐noise‐ratio (SNR) levels. For S (and equivalently OS and AS), NMEs were greater at lower *b*_t_ (particularly at low D*), whereas CoVs were greater at higher *b*_t_.
**Figure S3.** Simulated normalized root mean square errors (NRMSEs) of the estimated intravoxel incoherent motion (IVIM) pseudo‐diffusion parameters (D*, *f*, and *f* × D*) and molecular‐diffusion coefficient (*D*) using the segmented (S), oversegmented (OS), analytical segmented (AS), and optimized analytical segmented (opAS) fitting methods. The methods estimated D*, *f*, *f* × D*, and *D* at different combinations of *f* and D* at a fixed *D* value of 1.0 × 10^−3^ mm^2^/s. NRMSEs were measured with b‐threshold (*b*_t_) set to 100 s/mm^2^ and 200 s/mm^2^. In the plots, *f* increases vertically, and D* increases horizontally. Of the four methods, opAS had the lowest errors in the estimate of all the IVIM parameters at all regions and signal‐to‐noise‐ratio (SNR) levels, particularly at low D* values. Additionally, AS outperformed S and OS. For S, OS, and AS approaches, NRMSEs were higher at lower *b*_t_ (particularly at low D*). NRMSEs for all methods were greater at lower SNR levels (particularly at low *f*).
**Figure S4.** Subtracted contrast‐enhanced MRI (CE‐MRI) images and intravoxel incoherent motion (IVIM) parameter maps (*D*, molecular‐diffusion coefficient; D*, pseudo‐diffusion coefficient; *f*, pseudo‐diffusion fraction; and *f* × *D*_f_, pseudo‐diffusion flux) of the femoral heads of a second representative piglet fit using the four methods: segmented (S), oversegmented (OS), analytical segmented (AS), and optimized analytical segmented (opAS). CE‐MRI confirmed complete ischemia in the operated femoral heads (*yellow arrows*). For all the fitting methods, *D* increased and *f* and *f* × *D*_f_ decreased in the ischemic versus control femoral heads. However, there was a more pronounced decrease in D* in the ischemic femoral heads using the AS and opAS methods than using the S and OS approaches.
