# Supplementary material for: Optimized analytical segmented method for improved intravoxel incoherent motion parameter extraction in low‐perfused tissues
Source: Magn Reson Med. 2025 Jul 17;94(6):2508–18. doi: 10.1002/mrm.30636 (PMC12501690; doi:10.1002/mrm.30636)

**SUPPLEMENTAL INFORMATION**

**Figure S1:** Algorithmic steps used to extract IVIM parameters from diffusion-weighted MRI using the Segmented (S), Over Segmented (OS), Analytical Segmented (AS), and optimized Analytical Segmented (opAS) methods.


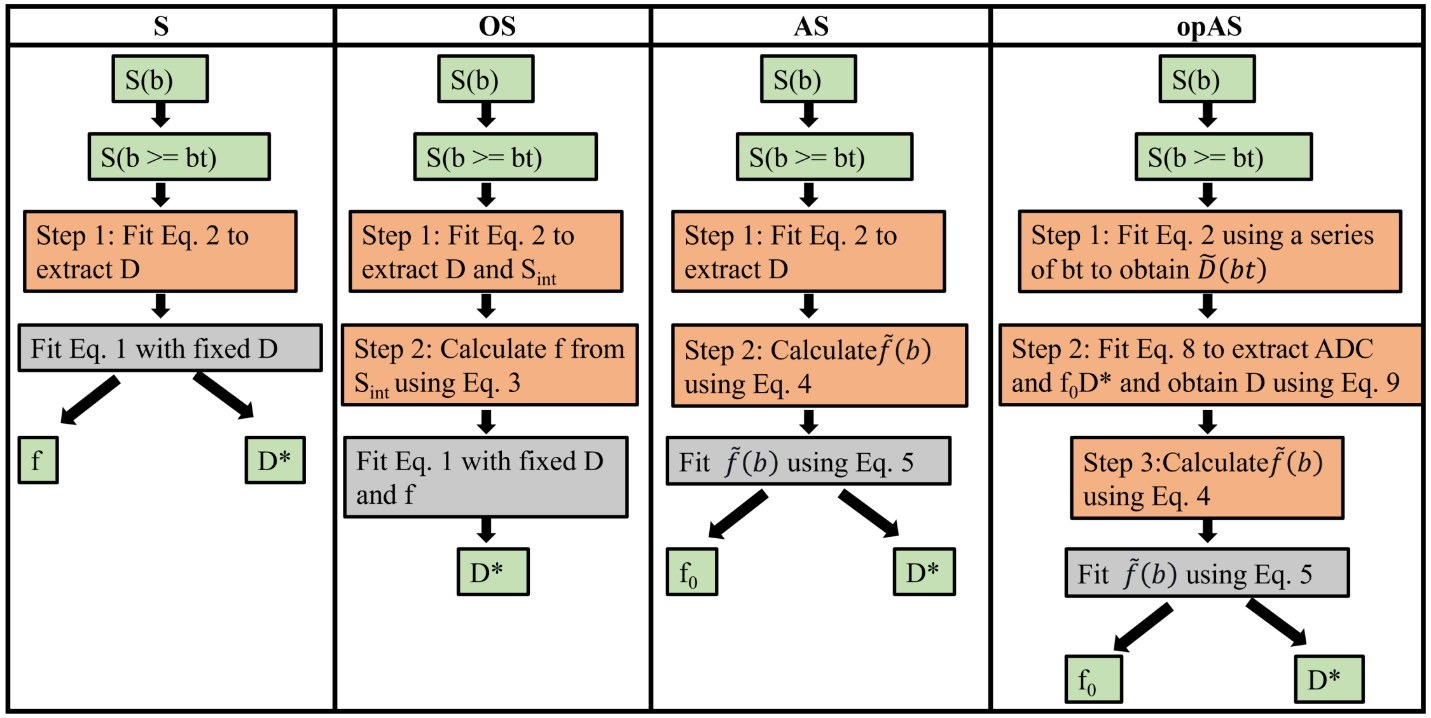


**Figure S2:** Simulated normalized mean errors (NMEs) and coefficients of variations (CoVs) of the estimated IVIM molecular diffusion coefficient (D) using the Segmented (S) (and equivalently Over Segmented (OS) and Analytical Segmented (AS)) and optimized Analytical Segmented (opAS) fitting methods. The methods estimated D at different combinations of f and D* at a fixed D value of 1.0×10^-3^ mm^2^/s. In the plots, f increases vertically, and D* increases horizontally with b-threshold (b_t_) set to 100 s/mm^2^ and 200 s/mm^2^. opAS had lower NMEs and CoVs in the estimate of D at all regions and SNR levels. For S (and equivalently OS and AS), NMEs were greater at lower b_t_ (particularly at low D*) while CoVs were greater at higher b_t_.


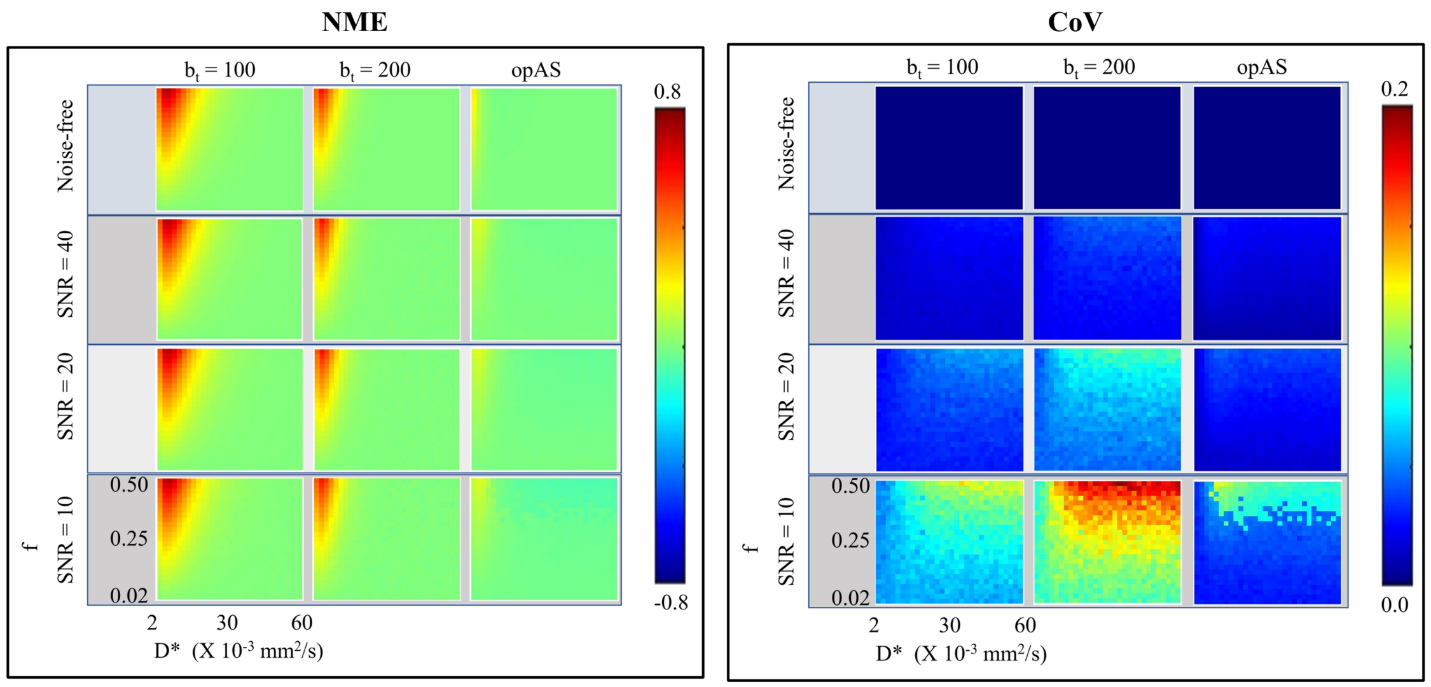


**Figure S3:** Simulated normalized root mean square errors (NRMSEs) of the estimated IVIM pseudo-diffusion parameters (D*, f, and f×D*) and molecular-diffusion coefficient (D) using the Segmented (S), Over Segmented (OS), Analytical Segmented (AS), and optimized Analytical Segmented (opAS) fitting methods. The methods estimated D*, f, f×D*, and D at different combinations of f and D* at a fixed D value of 1.0×10^-3^ mm^2^/s. NRMSEs were measured with b-threshold (b_t_) set to 100 s/mm^2^ and 200 s/mm^2^. In the plots, f increases vertically, and D* increases horizontally. Of the four methods, opAS had the lowest errors in the estimate of all the IVIM parameters at all regions and SNR levels, particularly at low D* values. Additionally, AS outperformed S and OS. For S, OS, and AS approaches, NRMSEs were higher at lower b_t_ (particularly at low D*). NRMSEs for all methods were greater at lower SNR levels (particularly at low f).


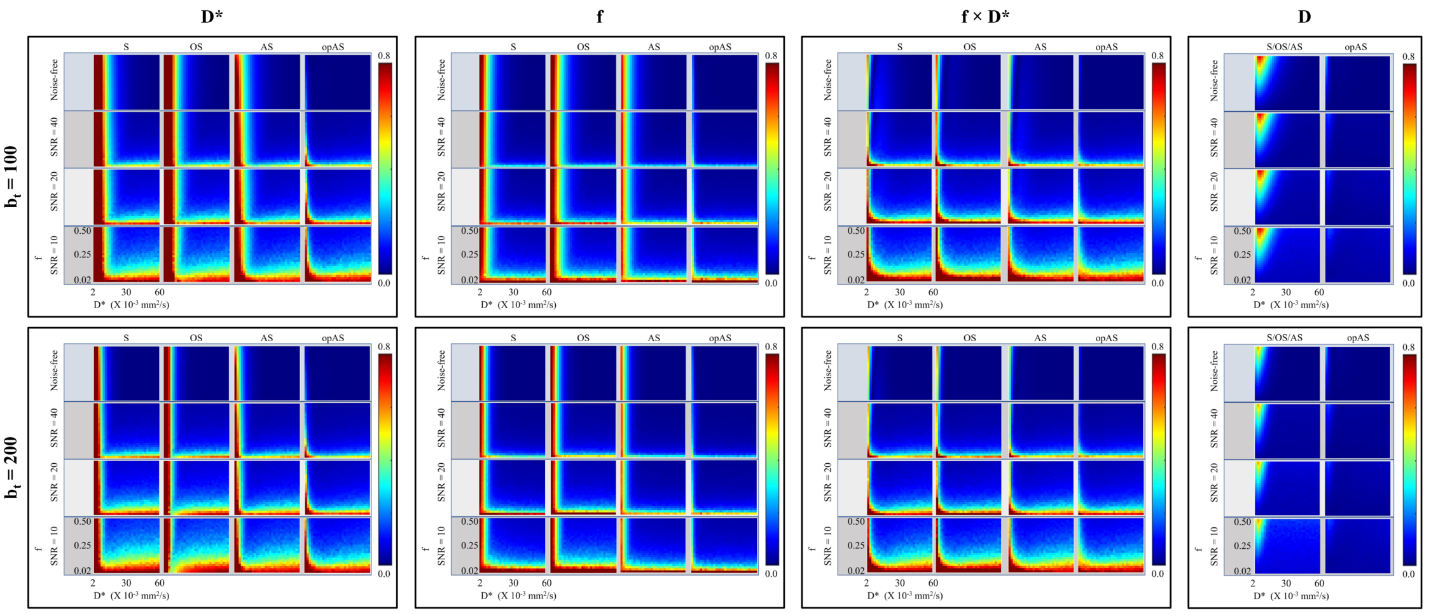


**Figure S4:** Subtracted contrast-enhanced MRI (CE-MRI) images and IVIM parameter maps (molecular-diffusion coefficient: D; pseudo-diffusion coefficient: D*; pseudo-diffusion fraction: f; and pseudo-diffusion flux: f×D_f_) of the femoral heads of a second representative piglet fit using the four methods: Segmented (S), Over Segmented (OS), Analytical Segmented (AS), and optimized Analytical Segmented (opAS). CE-MRI confirmed complete ischemia in the operated femoral heads (yellow arrows). For all of the fitting methods, D increased and f and f×D_f_ decreased in the ischemic vs. control femoral heads. However, there was a more pronounced decrease in D* in the ischemic femoral heads using the AS and opAS methods than using the S and OS approaches.


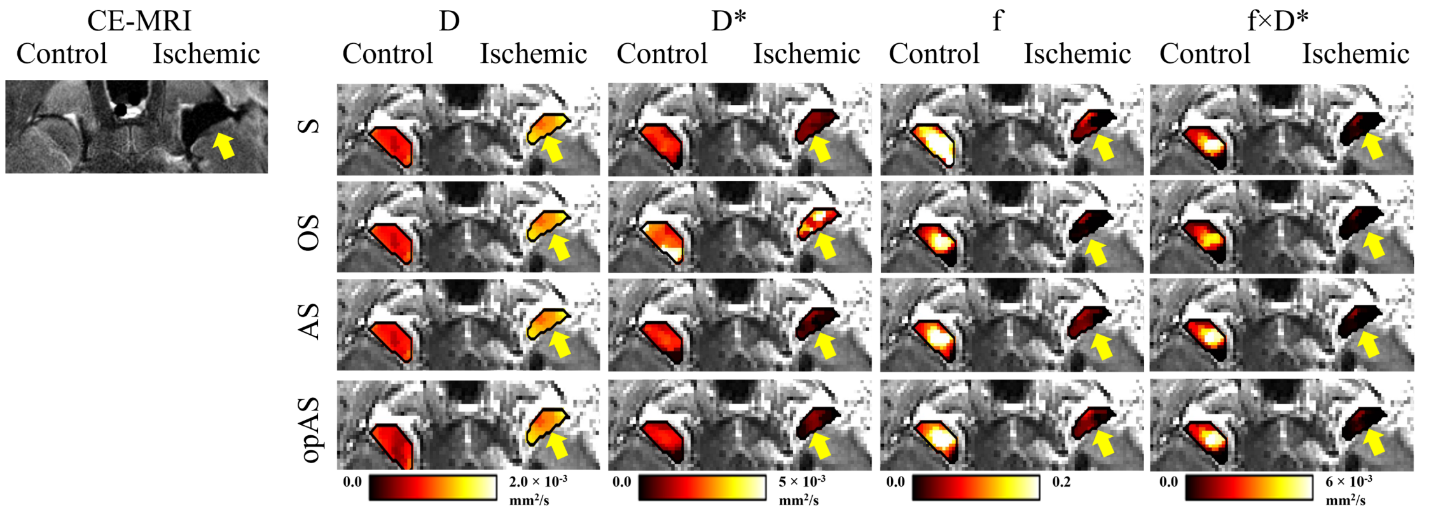

Supplement: Supplementary file 1 — Figure S1. Algorithmic steps used to extract intravoxel incoherent motion (IVIM) parameters from diffusion‐weighted MRI using the segmented (S), oversegmented (OS), analytical segmented (AS), and optimized analytical segmented (opAS) methods. Figure S2. Simulated normalized mean errors (NMEs) and coefficients of variation (CoVs) of the estimated intravoxel incoherent motion (IVIM) molecular diffusion coefficient (D) using the segmented (S) (and equivalently oversegmented [OS] and analytical segmented [AS]) and optimized analytical segmented (opAS) fitting methods. The methods estimated D at different combinations of f and D* at a fixed D value of 1.0 × 10^−3^ mm2/s. In the plots, f increases vertically, and D* increases horizontally with b‐threshold (b t) set to 100 s/mm2 and 200 s/mm2. The value of opAS had lower NMEs and CoVs in the estimate of D at all regions and signal‐to‐noise‐ratio (SNR) levels. For S (and equivalently OS and AS), NMEs were greater at lower b t (particularly at low D*), whereas CoVs were greater at higher b t. Figure S3. Simulated normalized root mean square errors (NRMSEs) of the estimated intravoxel incoherent motion (IVIM) pseudo‐diffusion parameters (D*, f, and f × D*) and molecular‐diffusion coefficient (D) using the segmented (S), oversegmented (OS), analytical segmented (AS), and optimized analytical segmented (opAS) fitting methods. The methods estimated D*, f, f × D*, and D at different combinations of f and D* at a fixed D value of 1.0 × 10^−3^ mm2/s. NRMSEs were measured with b‐threshold (b t) set to 100 s/mm2 and 200 s/mm2. In the plots, f increases vertically, and D* increases horizontally. Of the four methods, opAS had the lowest errors in the estimate of all the IVIM parameters at all regions and signal‐to‐noise‐ratio (SNR) levels, particularly at low D* values. Additionally, AS outperformed S and OS. For S, OS, and AS approaches, NRMSEs were higher at lower b t (particularly at low D*). NRMSEs for all methods were greater at lower [file MRM-94-2508-s001.docx]
